# Vascular response and intrastent thrombus in the early phase after drug‐eluting versus bare‐metal stent implantation in patients with ST‐segment elevation myocardial infarction: An observational, single‐center study

**DOI:** 10.1002/hsr2.105

**Published:** 2018-12-05

**Authors:** Nobuhiro Sato, Yoshiyasu Minami, Takao Shimohama, Ryo Kameda, Taiki Tojo, Junya Ako

**Affiliations:** ^1^ Department of Cardiovascular Medicine Kitasato University Hospital Sagamihara Japan

**Keywords:** acute coronary syndrome, dual antiplatelet therapy, neointimal coverage, optical coherence tomography

## Abstract

**Background and Objectives:**

Second‐generation drug‐eluting stents (G2‐DES) are associated with a lower rate of acute and subacute stent thrombosis compared with bare‐metal stent (BMS) in the setting of ST‐segment elevation myocardial infarction (STEMI). In this study, our aim was to compare the vascular response and thrombus burden between G2‐DES and BMS in early‐phase STEMI.

**Methods:**

Between May 2010 and August 2014, a total of 41 STEMI patients treated by either G2‐DES (n = 26; everolimus‐eluting stent [EES]: n = 15, zotarolimus‐eluting stent [ZES]: n = 11) or BMS (n = 15) and, with multivessel disease requiring additional percutaneous coronary intervention (PCI), were prospectively enrolled. Optical coherence tomography (OCT) imaging was performed at 1 month after stent implantation.

**Results:**

Baseline clinical characteristics, except for age (61.5 ± 9.3 vs 69.3 ± 9.8, *P* = 0.01, *t* test), were comparable between patients with drug‐eluting stent (DES) and BMS. The incidence of residual thrombus after the stent implantation for STEMI was comparable between DES and BMS (7.7% vs 6.7%, *P* = 0.88, *χ* ^2^ test). At 1 month, thrombus burden, defined as the mean thrombus area divided by the mean lumen area, was significantly smaller with DES than with BMS (median interquartile range (IQR), 1.2 (0.0, 1.0) vs 1.2 (0.0, 2.2), *P* = 0.04, Mann‐Whitney *U* test), despite a similar percentage of malapposed (median (IQR), 6.2 (2.4, 9.0) vs 2.6 (0.0, 5.8)%, *P* = 0.07, Mann‐Whitney *U* test) or uncovered struts (median (IQR), 6.8 (1.8, 13.1) vs 6.14 (2.8, 18.5)%, *P* = 0.45, Mann‐Whitney *U* test). No significant difference in thrombus burden was observed between EES and ZES.

**Conclusions:**

Thrombus burden was significantly smaller with DES than with BMS at 1‐month follow‐up in STEMI cases, although the percentage of malapposed or uncovered struts was similar between the groups. This may partly explain the lower rate of acute and subacute stent thrombosis in G2‐DES that has previously been reported in the literature.

## 1 INTRODUCTION

Although so‐called second‐generation drug‐eluting stents (G2‐DES) have been developed to overcome residual safety concerns from first‐generation drug‐eluting stent (DES),^1^, ^2^, ^3^, ^4^ delayed reendothelialization is still considered to be an inevitable safety matter in any type of DES.^5^, ^6^, ^7^, ^8^, ^9^, ^10^ In particular, the safety of DES in acute and subacute phases in cases of ST‐segment elevation myocardial infarction (STEMI) was debated as a result of the activated thrombogenicity,^11^, ^12^ instability of residual plaque,^13^ and higher frequency of suboptimal results in the procedure.^14^, ^15^ However, contrary to expectations, a lower rate of definite stent thrombosis with an everolimus‐eluting stent (EES) compared with the bare‐metal stent (BMS) was demonstrated in the EXAMINATION (clinical Evaluation of the Xience‐V stent in Acute Myocardial INfArcTION) trial.^16^ Surprisingly, the difference in the rate of thrombosis between the groups was most significant in acute and subacute phases. Although it may be favorable in all aspects of clinical practice, the mechanisms and factors underlying the fewer thrombotic events with G2‐DES compared with BMS in the early phase after acute myocardial infarction (AMI) remain unclear. Thus, the aim of this study was to compare the vascular response and thrombus burden between G2‐DES and BMS in early‐phase STEMI, using optical coherence tomography (OCT).

## 2 MATERIALS AND METHODS

### 2.1 Study population

**Figure 1:**
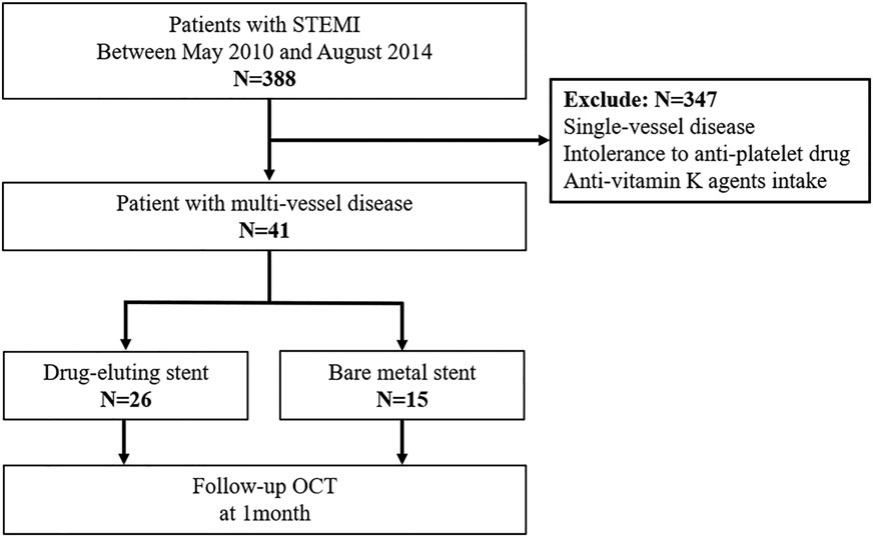
Study flowchart. OCT, Optical coherence tomography; STEMI, ST‐segment elevation myocardial infarction

This is an observational study conducted in a single center (Department of Cardiovascular Medicine, Kitasato University Hospital, Sagamihara, Japan). Between May 2010 and August 2014, a total of 388 consecutive patients with STEMI underwent percutaneous coronary intervention (PCI) with BMS or DES in our institute. Among them, a total of 41 patients with multivessel disease requiring additional PCI for nonculprit lesions were included in the present study. Culprit lesions (n = 48) were treated with EES (Xience, Abbott Vascular, Santa Clara, California: n = 15), zotarolimus‐eluting stent (E‐ZES; Endeavor, Medtronic, Minneapolis, Minnesota: n = 11), or BMS (n = 15) at the operator's discretion. Follow‐up OCT was performed at the time of PCI for nonculprit lesions 1 month after the STEMI (Figure 1). STEMI was defined as typical chest pain or discomfort lasting greater than 20 minutes, electrocardiogram showing new ST‐segment elevation greater than or equal to 0.2 mV in greater than or equal to two contiguous precordial leads, greater than or equal to 0.1 mV in greater than or equal to two contiguous limb leads, or new left bundle branch block, and cardiac markers (creatine kinase MB and cardiac troponin T or I) increased to greater than the upper reference.^17^ Cases with bypass graft lesions, stent thrombosis, and intolerance to antiplatelet drugs and contrast dye were excluded from the analysis. All patients provided written informed consent for all of the interventional procedures in the present study. The study protocol was approved by the ethics committee of our institution.

### 2.2 PCI procedure for STEMI

All patients underwent primary PCI within 24 hours after the onset of symptoms. All patients received aspirin (loading dose 200 mg and maintenance dose of 100 mg/d), clopidogrel (loading dose 300 mg and maintenance dose of 75 mg/d), and unfractionated heparin (5000‐IU bolus injection) before PCI. To keep an activated clotting time of greater than 250 seconds during the procedure, additional unfractionated heparin was administered accordingly. Thrombus aspiration, predilatation, and stent selection were left to the operator's discretion. Intravascular ultrasound (IVUS) was conducted in all cases to confirm optimal stenting without significant malapposition, underexpansion, or residual dissection before completing the procedure.

### 2.3 OCT image acquisition and analysis

**Figure 2:**
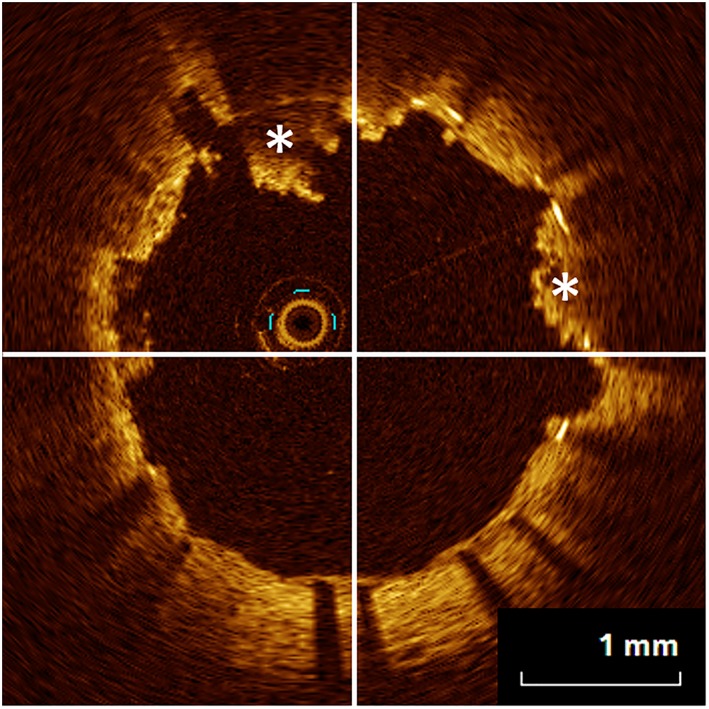
Optical coherence tomography (OCT) assessment of thrombus. A representative image of OCT cross section with thrombus is shown. Because thrombus (asterisks) are observed in the upper two quadrants, the score in this cross section would be 2. Thrombus score is calculated as the sum of each score through the stent

The OCT follow‐up was performed at 3 or 6 weeks after the initial PCI for STEMI. The follow‐up timing was completely dependent on the PCI for nonculprit lesions. OCT images were acquired using frequency domain (FD) OCT (C7‐XR OCT Intravascular Imaging System; St. Jude Medical Inc., St Paul, Minnesota). All images were analyzed using off‐line proprietary software (St. Jude Medical) by an investigator who was blinded to the stent type. The images were analyzed according to the consensus standards for acquisition and measurement of OCT.^18^ Quantitative analysis was performed at every 1‐mm interval. Neointimal hyperplasia (NIH) thickness was defined as the distance between the endoluminal surface of the neointima and the strut reflection: % NIH = (stent area − lumen area)/stent area × 100. The maximal incomplete stent apposition length was the distance between the endoluminal surface of the strut reflection and the vessel wall. The strut apposition was divided into four categories: (1) well‐apposed to the vessel wall with NIH, (2) well‐apposed to the vessel wall without NIH, (3) malapposed to the vessel wall with NIH, and (4) malapposed to the vessel wall without NIH. The covered strut is defined as a strut with surrounding tissue beyond the strut surface. The uncovered strut is defined as completely embedded with disruption of lumen contour or partially embedded with extension of strut into lumen.^19^, ^20^ Stent malapposition was defined as the center of the strut with detachment from the vessel wall greater than 20 μm plus thickness of stent strut: greater than 100 μm for Xience Prime, Xience Xpedition, and Multi‐link 8, greater than 110 μm for Integrity and Driver, and greater than 115 μm for Liberté. The well‐apposed coverage was calculated as well‐apposed struts with neointima divided by total struts. A thrombus was defined as an irregular protruding of signal‐rich, low‐backscattering protrusions or high‐backscattering protrusions beyond the stent struts into the lumen, with signal‐free shadowing and a sharp intensity gap with a dimension of greater than or equal to 250 μm on the OCT image.^21^, ^22^ Percent thrombus length was calculated as the thrombus length divided by the stent length. Thrombus burden was defined as the mean thrombus area divided by the mean lumen area. A semiquantitative assessment was also performed using the OCT‐thrombus score. A thrombus was classified as absent (0) or subtending 1, 2, 3, or 4 quadrants in each cross section.^23^ Thrombus score was calculated as the sum of each score (Figure 2). In the evaluation of apposition and NIH, overlapping stents and bifurcation lesions with major side branches were excluded from the analysis. Evaluation of % thrombus includes overlapping and bifurcation lesions with major side branches. Other definitions are described in the Supporting Information.

### 2.4 Statistical methods

Categorical variables were reported as counts (%) and compared by the *χ* ^2^ test. The average with standard deviation was reported when the data were normally distributed, and the median with interquartile range was reported when the data were not normally distributed. Continuous variables were compared by the *t* test when the data were normally distributed and compared by the Mann‐Whitney *U* test when the data were not normally distributed. All tests were two‐sided, and statistical significance was defined as *P* < 0.05. All statistical analyses were performed using JMP 9.0 version (SAS Institute, Cary, North Carolina).

## 3 RESULTS

### 3.1 Patient characteristics

There were no significant differences in baseline clinical characteristics between the DES group and the BMS group other than a higher age in the BMS group (Table 1). Baseline lesion and procedural characteristics including the incidence of residual thrombus evaluated by final IVUS images after the stent implantation were comparable between patients with DES and BMS (Table 2). Medications at discharge are shown in Table 3. All those medications were prescribed with the same dose and type until follow‐up.

**Table 1:** Baseline clinical characteristics

	DES (N = 26)	BMS (N = 15)	*P* Value
Age, y	61.5 ± 9.3	69.3 ± 9.8	0.01^a^
Male, n (%)	25 (96)	12 (80)	0.26^b^
BMI, kg/m^2^	25.7 ± 3.7	26.8 ± 6.1	0.46^a^
Risk factors, n (%)			
Diabetes mellitus	9 (35)	6 (40)	0.73^b^
Hypertension	14 (54)	12 (80)	0.18^b^
Dyslipidemia	18 (69)	11 (73)	0.78^b^
Renal dysfunction	10 (38)	4 (27)	0.44^b^
Current smoking	15 (58)	5 (33)	0.13^b^
Prior MI	0	0	‐
Prior PCI	0	0	‐
Prior CABG	0	0	‐
Onset to door time, h (median (IQR))	1.2 (0.8, 2.9)	1.4 (1.4, 1.7)	0.26^c^
Killip III/IV, n (%)	5 (19)	0 (0)	0.19^b^
Laboratory data (mean ± SD or median (IQR))			
Triglyceride, mg/dL	108.6 ± 53.2	113.6 ± 56.0	0.78^a^
LDL‐C, mg/dL	122.7 ± 37.8	105.7 ± 32.7	0.15^a^
HbA_1c_, %	6.2 ± 0.8	6.9 ± 1.7	0.06^a^
Creatinine, mg/dL	0.9 ± 0.2	0.9 ± 0.3	0.57^a^
Peak CK, μg/L	2166 ± 1546	1493 ± 1671	0.19^a^
Peak CK‐MB, μg/L	225 ± 184	139 ± 158	0.13^a^
TnI, ng/mL	0.06 (0.03, 0.70)	0.7 (0.06, 2.89)	0.18^c^
LVEF, %	49.5 ± 14.3	57.6 ± 9.5	0.06^a^

Abbreviations: BMI, body mass index; BMS, bare‐metal stent; CABG, coronary artery bypass graft; CK‐MB, creatine kinase myocardial band; DES, drug‐eluting stent; LDL‐C, low‐density lipoprotein cholesterol; LVEF, left ventricular ejection fraction on echocardiography; MI, myocardial infarction; PCI, percutaneous coronary intervention; SD, standard deviation; TnI, troponin I.

a *t* test.

b *χ* ^2^ test.

c Mann‐Whitney *U* test.

**Table 2:** Baseline lesion and procedural characteristics

	DES (N = 26)	BMS (N = 15)	*P* Value
Door to balloon time, h (mean ± SD)	1.3 ± 0.3	1.5 ± 0.4	0.06^a^
Lesion location			0.38^b^
LAD, n (%)	10 (38)	4 (27)	
RCA, n (%)	12 (46)	11 (73)	
LCX, n (%)	4 (15)	0 (0)	
Left main, n (%)	0 (0)	0 (0)	
Initial TIMI flow grade			1.00^b^
0/1, n (%)	26 (100)	15 (100)	
2/3, n (%)	0 (0)	0 (0)	
Manual thrombectomy, n (%)	21 (81)	9 (60)	0.15^b^
Predilatation, n (%)	17 (65)	10 (67)	0.93^b^
IVUS guide, n (%)	26 (100)	15 (100)	1.00^b^
Stent diameter, mm (mean ± SD)	3.1 ± 0.3	3.2 ± 0.4	0.32^a^
Stent length, mm (mean ± SD)	29.2 ± 16.0	20.4 ± 7.6	0.05^a^
Post dilatation, n (%)	18 (69)	7 (47)	0.15^b^
Final TIMI 3 flow, n (%)	26 (100)	15 (100)	1.00^b^
IVUS findings			
MSA, mm^2^ (mean ± SD)	6.2 ± 1.6	6.5 ± 1.8	0.58^a^
Thrombus, n (%)	2 (7.7)	1 (6.7)	0.88^b^

Abbreviations: BMS, bare‐metal stent; DES, drug‐eluting stent; IVUS, intra vascular ultrasound; LAD, left anterior descending artery; LCX, left circumflex artery; MSA, minimal stent area; RCA, right coronary artery; SD, standard deviation; TIMI, thrombolysis in myocardial infarction.

a *t* test.

b *χ* ^2^ test.

**Table 3:** Medication at discharge

	DES (N = 26)	BMS (N = 15)	*P* Value^a^
Drugs, n (%)			
ACE inhibitor	22 (85)	9 (60)	0.16
Angiotensin II receptor blocker	4 (15)	5 (33)	0.34
Calcium‐channel inhibitor	1 (3.8)	1 (6.7)	0.67
Statin	26 (100)	15 (100)	1.00
Clopidogrel	26 (100)	15 (100)	1.00
Aspirin	26 (100)	15 (100)	1.00

Abbreviations: ACE, angiotensin‐converting enzyme; BMS, bare‐metal stent; DES, drug‐eluting stent.

a *χ* ^2^ test.

### 3.2 NIH at 1 month

A total of 966 cross sections and 9871 struts were analyzed (Table 4). No significant difference was observed between the DES group and the BMS group in the percentage of struts with neointimal coverage irrespective of the apposition status. The DES group had significantly smaller % NIH and thinner NIH than the BMS group (5.3 ± 2.0% vs 10.1 ± 7.3%, *P* < 0.01, 65.4 ± 16.4 vs 133.7 ± 93.6 μm, *P* < 0.01, *t* test, respectively).

**Table 4:** Follow‐up OCT findings

	DES (N = 26)	BMS (N = 15)	*P* Value
Follow‐up days (mean ± SD)	31.6 ± 10.5	28.5 ± 10.3	0.31^a^
Stent‐level analysis			
Thrombus, n (%)	11 (42)	9 (60)	0.28^b^
Tissue protrusion, n (%)	11 (42)	7 (47)	0.78^b^
Smooth protrusion, n (%)	7 (27)	0 (0)	0.02^b^
Irregular protrusion, n (%)	6 (23)	7 (47)	0.12^b^
Stent edge dissection, n (%)	2 (7.7)	1 (6.7)	0.90^b^
Major, n (%)	2 (7.7)	0 (0)	0.27^b^
Minor, n (%)	0 (0)	1 (6.7)	0.18^b^
Strut‐level analysis	N = 6666	N = 3205	
Well‐apposed, % (median (IQR))	93.4 (89.0, 97.6)	100.0 (88.4, 100.0)	0.08^c^
With neointimal, %	85.2 (80.4, 90.5)	87.7 (78.0, 95.0)	0.58^c^
Without neointimal, %	6.3 (1.5, 13.1)	5.6 (2.8, 15.0)	0.93^c^
Malapposed, % (median (IQR))	6.2 (2.4, 9.0)	2.6 (0.0, 5.8)	0.07^c^
With neointimal, %	6.2 (2.2, 8.3)	1.9 (0.0, 6.4)	0.10^c^
Without neointimal, %	0.1 (0.0, 0.9)	0.0 (0.0, 1.3)	0.72^c^
% NIH (mean ± SD)	5.3 ± 2.0	10.1 ± 7.3	<0.01^a^
Mean NIH thickness, μm (mean ± SD)	65.4 ± 16.4	133.7 ± 93.6	<0.01^a^
Max malapposed length, μm (median (IQR))	330 (200, 510)	0 (0.0, 470)	0.05^c^

Abbreviations: BMS, bare‐metal stent; DES, drug‐eluting stent; NIH, neointimal hyperplasia; OCT, optical coherence tomography; SD, standard deviation. A total of 966 cross sections and 9871 struts were analyzed.

a *t* test.

b *χ* ^2^ test.

c Mann‐Whitney *U* test.

### 3.3 Thrombus at 1 month

**Figure 3:**
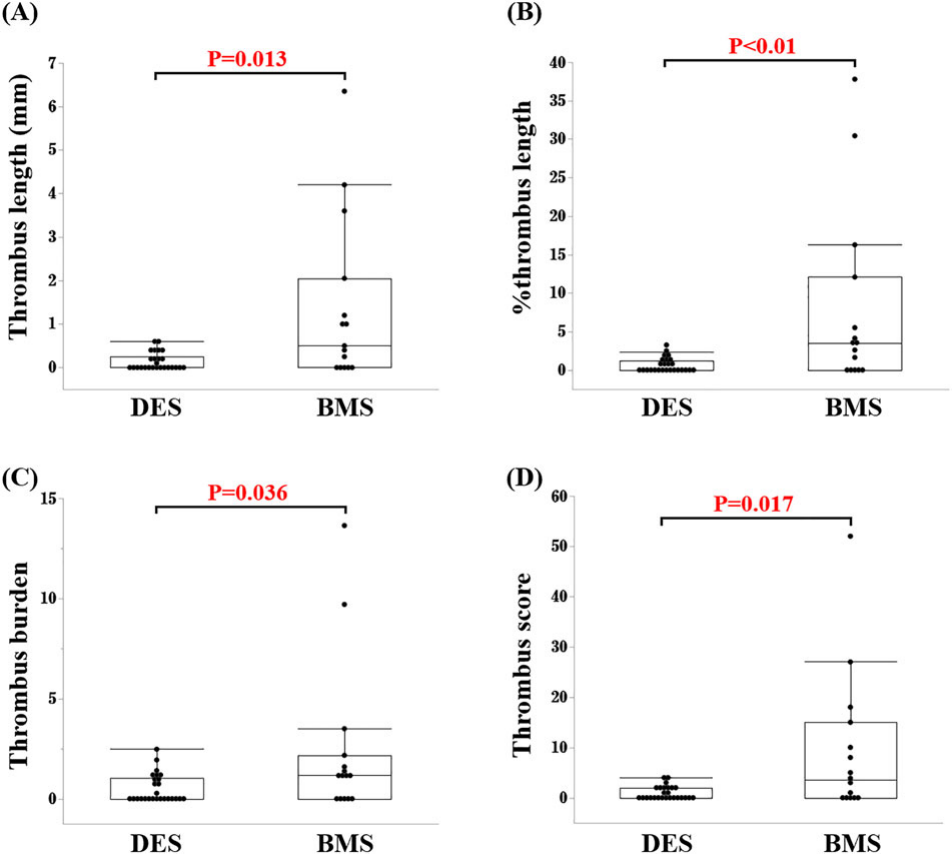
Optical coherence tomography (OCT) assessment of thrombus at 1 mo. Box plots show the following: A, Thrombus length. B, Percent thrombus length was calculated as the thrombus length divided by the stent length. C, Thrombus burden was defined as the mean thrombus area divided by the mean lumen area. D, Thrombus score was the sum of each score classified as absent (0) or subtending 1, 2, 3, or 4 quadrants in each cross section. BMS, bare‐metal stent; DES, drug‐eluting stent. *P* value was calculated by Mann‐Whitney *U* test. The box represents the interquartile range and the line in the box, the median. Whiskers represent 1.5 IQR

**Figure 4:**
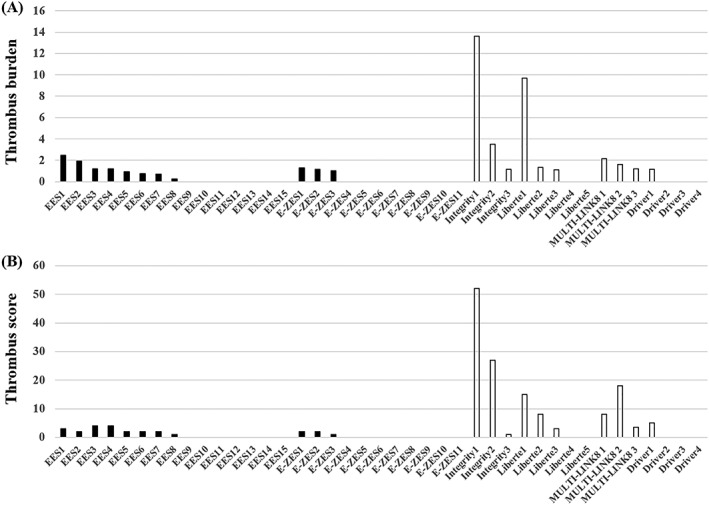
Thrombus assessment in each individual stent. Bar plot shows the following: A, Thrombus burden was defined as the mean thrombus area divided by the mean lumen area. B, Thrombus score was the sum of each score classified as absent (0) or subtending 1, 2, 3, or 4 quadrants in each cross section. EES, everolimus‐eluting stent; E‐ZES, zotarolimus‐eluting stent

**Figure 5:**
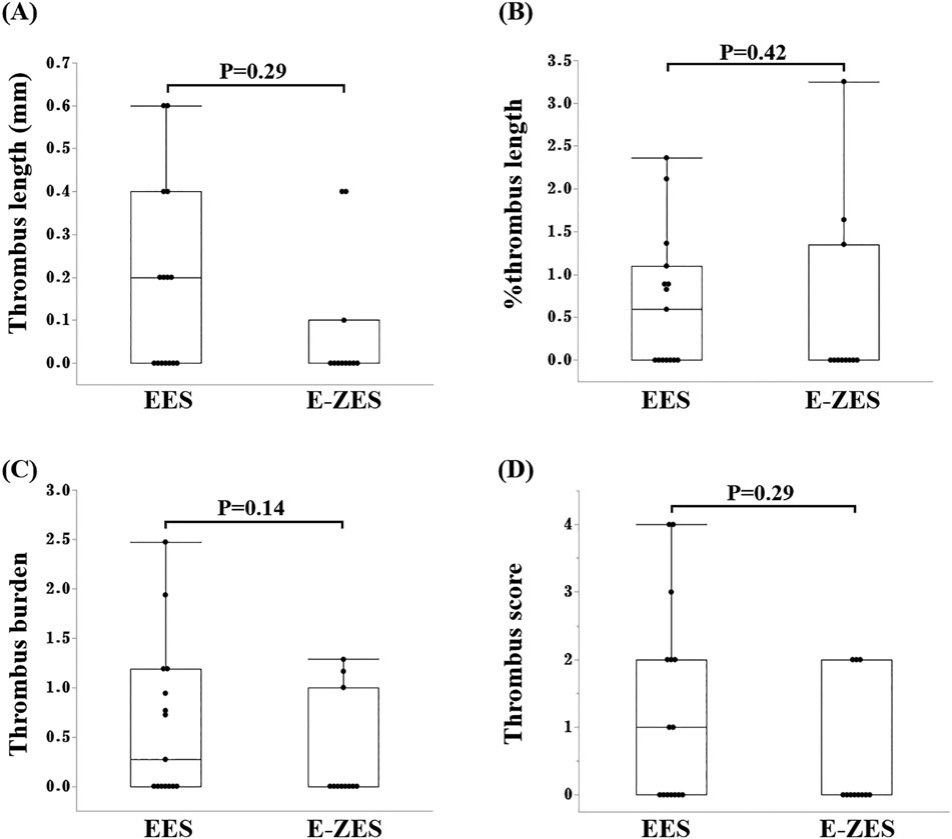
Comparisons of thrombus between EES and E‐ZES. Box plots show the following: A, Thrombus length. B, Percent thrombus length was calculated as the thrombus length divided by the stent length. C, Thrombus burden was defined as the mean thrombus area divided by the mean lumen area. D, Thrombus score was the sum of each score classified as absent (0) or subtending 1, 2, 3, or 4 quadrants in each cross section. EES, everolimus‐eluting stent; E‐ZES, zotarolimus‐eluting stent. *P* value was calculated by Mann‐Whitney *U* test. The box represents the interquartile range and the line in the box, the median. Whiskers represent 1.5 IQR

Thrombus length, % thrombus length, thrombus burden, and thrombus score were significantly smaller in the DES group than in the BMS group (median (IQR), 0.0 (0.0, 0.3) vs 0.5 (0.0, 2.1) mm, *P* = 0.013; 0.0 (0.0, 1.2) vs 3.5 (0.0, 12.1)%, *P* < 0.01; 0.0 (0.0, 1.0) vs 1.2 (0.0, 2.2), *P* = 0.036; 0.0 (0.0, 2.0) vs 3.5 (0.0, 15.0), *P* = 0.017, Mann‐Whitney *U* test, respectively) (Figure 3). Thrombus burden and thrombus score with the individual stent are shown in Figure 4. There was no significant difference in thrombus burden and thrombus score between the EES and the E‐ZES (Figure 5).

## 4 DISCUSSION

The main findings of the present study are the following: (1) Thrombus burden and thrombus score were significantly smaller in DES than in BMS at 1‐month follow‐up in STEMI patients, (2) the rate of malapposed and uncovered struts was comparable between DES and BMS at the 1‐month follow‐up, and (3) no significant differences in thrombus burden or the rate of uncovered struts was observed between EES and E‐ZES at 1‐month follow‐up.

### 4.1 Thrombogenicity and stent characteristics

The causes of thrombus formation and subsequent clinical events after coronary stent implantation are considered to be the accumulation of unfavorable factors in patient (eg, diabetes and hereditary drug withdrawal), procedural (eg, suboptimal stenting), device (eg, metal and polymer), lesion (eg, residual thrombus and lipid‐rich plaque), and pharmacological responses to antiplatelet drugs.^24^, ^25^, ^26^, ^27^, ^28^ Renewed technologies in G2‐DES such as biocompatible polymers, improved drug‐eluting kinetics, thinner struts, and a well‐designed platform with better conformability may contribute to the reduction of thrombus formation and clinical events compared with first‐generation DES and BMS.^1^, ^2^, ^3^, ^4^ Although the exact mechanism of smaller thrombus burden in G2‐DES compared with BMS in the present study still remains unclear, the presence of biocompatible polymer in G2‐DES might play an important role as a protective factor for thrombus formation, among several differences between them. The polymer in DES is a carrier of eluted drug and has several characteristics that improve biocompatibility of the stent through anti‐inflammatory and antithrombotic effects.^29^, ^30^, ^31^, ^32^ Previous studies demonstrated that polymer coating provides thromboresistance through the modification of surface properties such as electrostatic forces, hydrophilic interaction, and roughness.^33^, ^34^, ^35^ In particular, the fluoropolymer in Xience‐EES was designed to have better biocompatibility for blood and vascular tissues compared with that in older stents.^36^, ^37^, ^38^ Another study investigating vascular responses to Xience‐EES early after the implantation in STEMI also demonstrated a decreased thrombus volume, although the authors mentioned that the combination of multiple factors, including biocompatible polymer, contributed to the favorable vascular behavior.^39^ These results may alleviate the safety concern for a shorter duration of dual antiplatelet therapy in patients receiving Xience‐EES; this is currently being tested in a clinical trial named STOPDAPT‐2 (ShorT and OPtimal Duration of Dual AntiPlatelet Therapy‐2) (ClinicalTrials.gov identifier: NCT02619760).

From findings in previous studies, strut thickness is considered to be an important factor for the thrombogenicity of coronary stents.^40^ However, we think that the impact of strut thickness on the results in the present study was limited because we found a difference in thrombus burden among stents having a similar platform with thin struts. In addition to stent characteristics, vascular responses including neointimal coverage, strut malapposition, tissue protrusion,^13^ and dissection may cause focal thrombus formation and subsequent clinical events, particularly in patients with acute coronary syndrome.^41^ However, in the present study, the incidence of those findings on OCT images was comparable between the groups. Taken together, the presence of polymer in G2‐DES might be the major contributor, among various others, to the smaller thrombus burden compared with BMS in the early phase after implantation.

### 4.2 Thrombogenic properties among DES types

A significant difference in thrombus burden was not observed between EES and E‐ZES in the present study, although previous studies have demonstrated a difference in thrombogenic properties among different types of DES. In a recent study using a swine shunt model, Xience‐EES showed the best performance in terms of antithrombogenic properties compared with BioMatrix Flex‐BES, Synergy‐EES, Nobori‐BES, and Orsiro‐SES.^29^ Among several characteristics of Xience‐EES, the circumferential coating of the polymer may play a key role in decreasing thrombus formation.^29^, ^40^ An abluminal polymer coating in BioMatrix Flex‐BES and Synergy‐EES may have a disadvantage in terms of thromboresistance via the inhibition of platelet aggregation, because the bare‐metal surface is exposed to blood flow. From findings in the swine shunt model study, we should also focus on the effect of the material in the polymer. Although Orsiro‐SES has a circumferential polymer in addition to the thinner strut than that in the Xience‐EES, the result was worse in Orsiro compared with Xience‐EES.^29^ In the Orsiro stent, the polymer was composed of a hybrid coating with passive PROBIO amorphous silicon carbide (Biotronik) and active BIOlute bioabsorbable poly‐l‐lactide acid coating (Biotronik). In contrast, the polymer in Xience‐EES was poly(*n*‐butyl methacrylate) (PBMA) encapsulated by a poly(vinylidene fluoride‐co‐hexafluoropropylene) (PVDF‐HFP). PVDF‐HFP (used on Xience‐EES) retained more albumin and platelet interaction behavior than PBMA and polystyrene‐*b*‐polyisobutylene‐*b*‐polystyrene (used on Taxus Liberté‐PES).^32^ Thus, the difference in protein and surface materials may be attributed to the different properties in antithrombogenicity between the two DES. The polymer in E‐ZES was 2‐methacryloyloxyethyl phosphorylcholine (MPC). MPC could significantly decrease platelet adhesion and prolong clotting time because the tightly bound water layer forms a physical and energetic barrier to prevent protein adsorption and platelet adhesion on the surface.^42^, ^43^, ^44^ Thus, the antithrombogenic property of E‐ZES might be equivalent to EES. In fact, there was no significant difference in acute or subacute thrombosis between DES and ZES at 1‐year follow‐up in a meta‐analysis of clinical trials.^45^

### 4.3 Limitations

Several limitations in this study require acknowledgment. First, this is a nonrandomized observational study conducted in a single center with a limited number of patients. Second, as an observational study, a formal sample size calculation was not performed, and thus, the study may be underpowered. Third, the residual thrombus was not volumetrically evaluated at the end of the primary procedure by OCT. Differences in residual thrombus volume among groups might have affected the results.

## 5 CONCLUSION

Thrombus burden was significantly smaller with DES than with BMS at 1‐month follow‐up in STEMI cases, although the percentage of uncovered struts was similar between the groups. This may partly explain the lower rate of acute and subacute stent thrombosis in G2‐DES that has been shown in previous studies.

## CONFLICTS OF INTEREST

Yoshiyasu Minami received lecture fees from Abbott vascular. Junya Ako received lecture fees from Abbott vascular. These conflicts did not affect the study design, analysis, or interpretation.

## DISCLOSURE OF INTERESTS

All authors meet the authorship criteria according to the latest guidelines of the International Committee of Medical Journal Editors. All authors are in agreement with the present study. The authors had full access to all of the data in this study and take complete responsibility for the integrity of the data and the accuracy of the data analysis.

## AUTHOR CONTRIBUTIONS

Conceptualization: Nobuhiro Sato, Yoshiyasu Minami, Takao Shimohama, Ryo Kameda, Taiki Tojo, Junya Ako

Data Curation: Nobuhiro Sato

Formal Analysis: Nobuhiro Sato, Yoshiyasu Minami

Investigation: Nobuhiro Sato

Writing—Original Draft Preparation: Nobuhiro Sato

## Supporting information

Data S1. Supporting information
