# Supplementary material for: Vascular response and intrastent thrombus in the early phase after drug‐eluting versus bare‐metal stent implantation in patients with ST‐segment elevation myocardial infarction: An observational, single‐center study
Source: Health Sci Rep. 2018 Dec 5;2(1):e105. doi: 10.1002/hsr2.105 (PMC6346990; doi:10.1002/hsr2.105)
Supplement: Supplementary file 1 — Data S1. Supporting information [file HSR2-2-e105-s001.docx]

**SUPPLEMENTAL MATERIAL**

**Vascular Response and Intrastent Thrombus in the Early Phase After Drug-eluting Versus Bare Metal Stent Implantation in Patients with ST-segment Elevation Myocardial Infarction: an observational, single-center study**

**Brief title:** Thrombus burden in early phase after STEMI

Nobuhiro Sato, MD, Yoshiyasu Minami, MD, PhD, Takao Shimohama, MD, PhD, Ryo Kameda, MD, PhD, Taiki Tojo, MD, PhD, Junya Ako, MD, PhD

**Supplemental material**

- **Supplemental methods**
- **Supplemental references**

**Supplemental methods**

***Definitions of post stent vessel injuries***

Instent tissue protrusion was divided into 2 categories: smooth protrusion and irregular protrusion [1]. Smooth protrusion was defined as the bowing of plaque into the lumen between stent struts, without intimal disruption, appearing as a smooth semicircular arc connecting adjacent struts, and likely representing compression of soft plaque by the stent. Irregular protrusion was defined as protrusion of material with an irregular surface with maximal height ≥100 μm into the lumen between stent struts.

Stent edge dissection was defined as the disruption of the vessel luminal surface with a visible flap at the stent edge or 5 mm proximal and distal reference segments [1]. Major dissection was defined as the dissected flap with greater than 60 degrees in arc on cross sectional OCT image and/or 3mm in length on longitudinal OCT image. Other stent edge dissection was classified as minor [2].

**Supplemental references**

[1] Soeda T, Uemura S, Park S-J, Jang Y, Lee S, Cho J-M, et al. Incidence and Clinical Significance of Poststent Optical Coherence Tomography FindingsCLINICAL PERSPECTIVE. Circulation 2015;132:1020–9.

[2] Ali ZA, Maehara A, Généreux P, Shlofmitz RA, Fabbiocchi F, Nazif TM, et al. Optical coherence tomography compared with intravascular ultrasound and with angiography to guide coronary stent implantation (ILUMIEN III: OPTIMIZE PCI): a randomised controlled trial. Lancet. 2016;388:2618-28.
